# Supplementary material for: HIF-1β Positively Regulates NF-κB Activity via Direct Control of TRAF6
Source: Int J Mol Sci. 2020 Apr 24;21(8):3000. doi: 10.3390/ijms21083000 (PMC7216149; doi:10.3390/ijms21083000)
Supplement: Supplementary file 1 [file ijms-21-03000-s001.pdf]

Supplementary Figures and Legends-D'Ignazio et al.

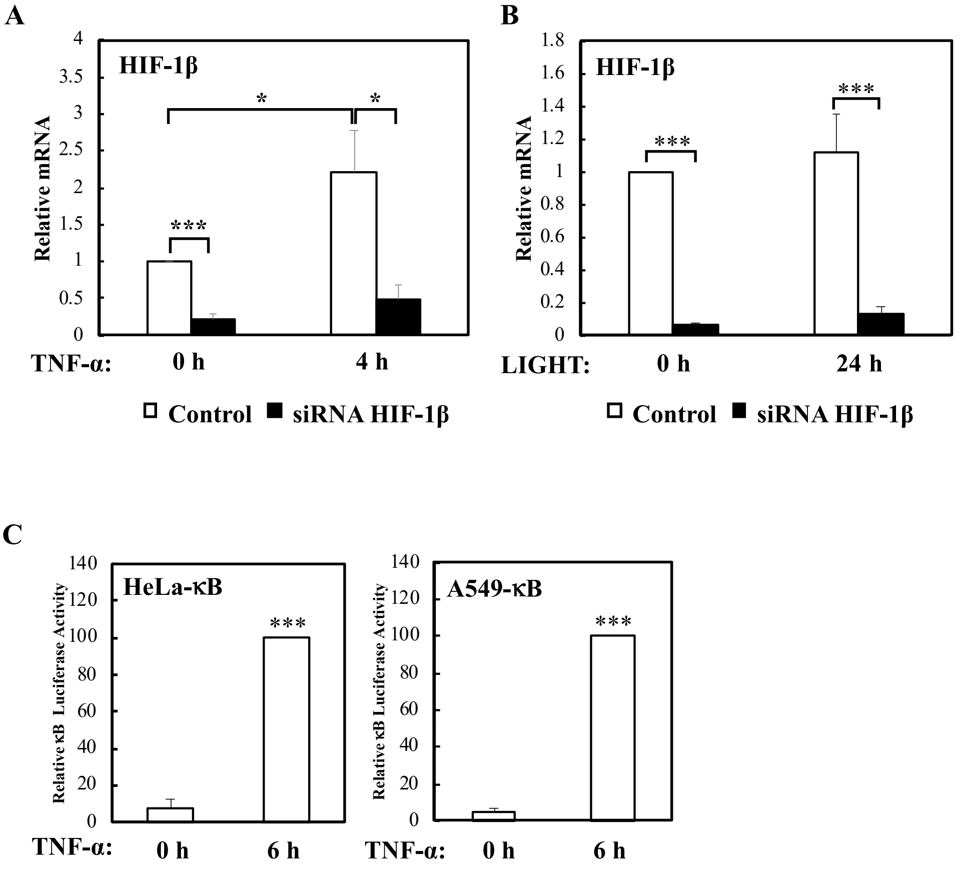

Sup. Fig S1. Control experiments for HeLa and A549 cells. A. HeLa cells were transfected with control or HIF-1β siRNA oligonucleotides before treatment, where indicated, with 10ng/mL TNF-α for 4 hours prior to lysis and RNA extraction. Following cDNA synthesis, qPCR was performed using HIF-1β specific primers. Graph depicts mean and SEM of a minimum of three independent biological experiments. One-way ANOVA was performed and significance determined as follows: *$p<0.05$, ***$p<0.001$. **B.** HeLa cells were transfected with control or HIF-1β siRNA oligonucleotides before treatment, where indicated, with 100ng/mL LIGHT for 24 hours prior to lysis and RNA extraction. Following cDNA synthesis, qPCR was performed using HIF-1β specific primers. Graph depicts mean and SEM of a minimum of three independent biological experiments. One-way ANOVA was performed and significance determined as follows: ***$p<0.001$. **C.** Characterisation of HeLa-κB and A549-κB luciferase cells response to 10ng/mL TNF-α. Values were normalised to TNF-α treated samples. Graphs

depict mean and SEM of a minimum of three independent biological experiments. Student t-test was performed and significance determined as follows: ***p<0.001.

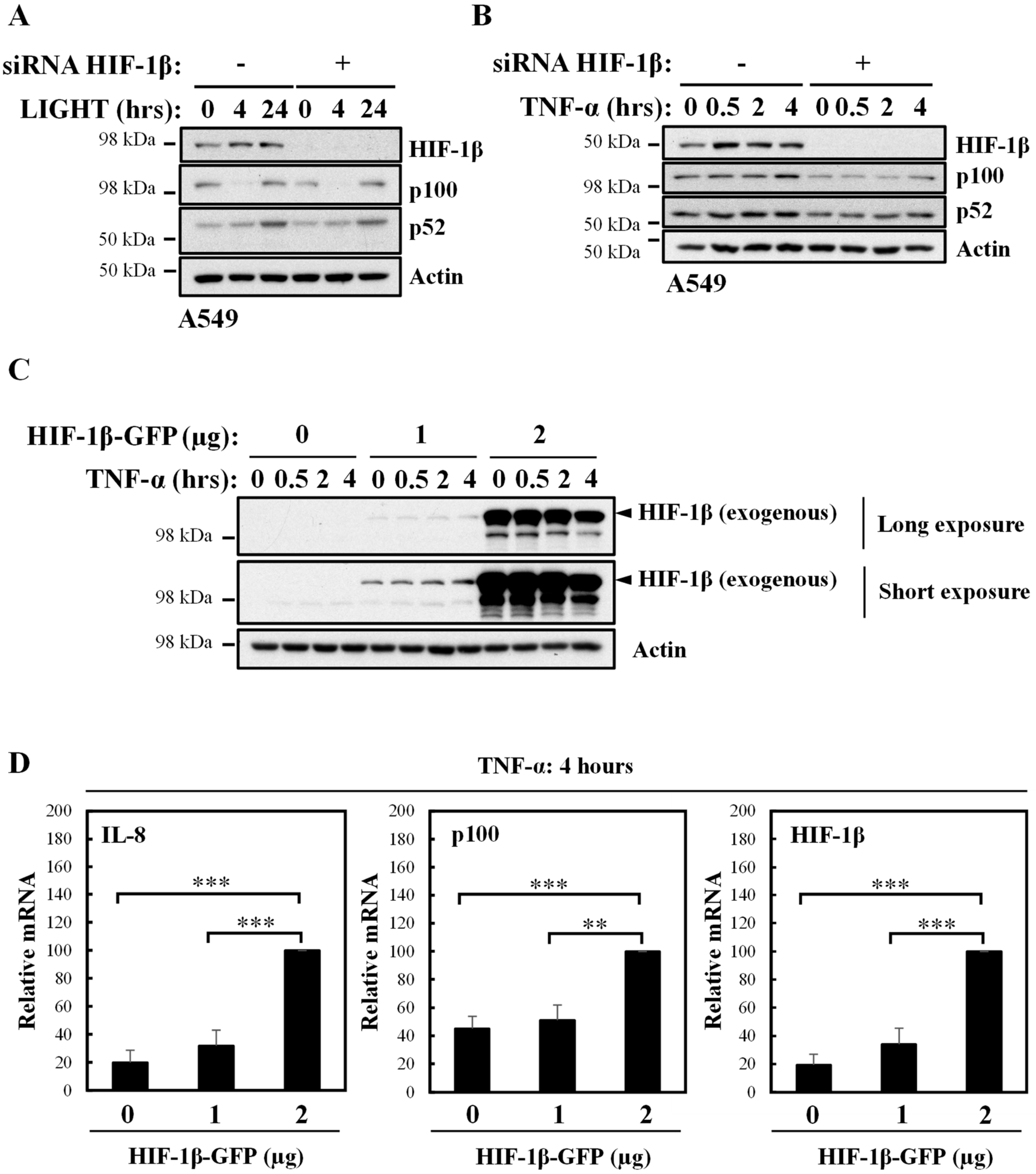

Sup. Fig. S2. HIF-1β levels control NF-κB transcriptional responses. A. A549 cells were transfected with control or HIF-1β siRNA oligonucleotides before treatment with 100ng/mL LIGHT for the indicated periods of time prior to lysis and Western blot analysis. Actin was used as a loading control. **B.** A549 cells were transfected with control or HIF-1β siRNA oligonucleotides before treatment with 10ng/mL TNF-α for the indicated periods of time prior to lysis and Western blot analysis. Actin was used as a loading control. **C.** HeLa cells were

transfected with 2 μg of control plasmid or the indicated amounts of HIF-1β plasmids, and treated with 10ng/mL TNF-α for the indicated times prior to lysis and Western blot analysis. Actin was used as a loading control. **D.** HeLa cells were transfected and treated as in *C*, but RNA was extracted. Following cDNA synthesis, qPCR was performed using the specific primers indicated. Graphs depict mean and SEM of a minimum of three independent biological experiments. One way ANOVA analysis was performed and significance determined as follows: **p<0.01; ***p<0.001.

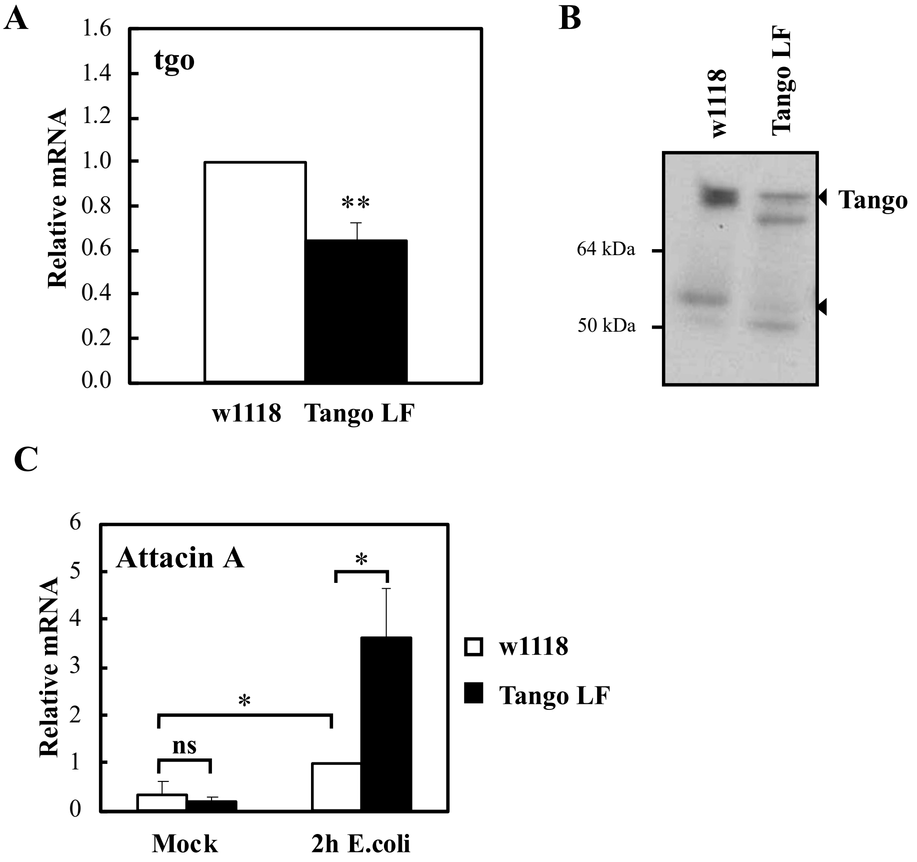

Sup. Fig. S3. ***Drosophila* HIF-1β expression in Tango loss-of-function flies. A.** Total mRNA extraction was performed on adult wild-type (w1118), and Tango loss-of-function flies (Tango LF), in conditions of normal development and homeostasis. Levels of Tango transcripts (tgo) were analysed using Actin as a normalising gene. The graph depicts average and SEM of three independent biological experiments. Student t-test was performed, and significance determined as follows: ** $p \leq 0.01$. **B.** Tango protein level in w1118 (control) and Tango loss-of-function (Tango LF) adult flies. A truncated form of protein in Tango LF flies was detected. **C.** Total mRNA extraction was performed on adult wild-type (w1118), and Tango loss-of-function flies (Tango LF), in conditions of mock (PBS) or *E. coli* infection. Levels of Attacin A transcript

were analysed using Actin as a normalising gene. The graph depicts average and SEM of three independent biological experiments. Student t-test was performed, and significance determined as follows: * $p \leq 0.05$.

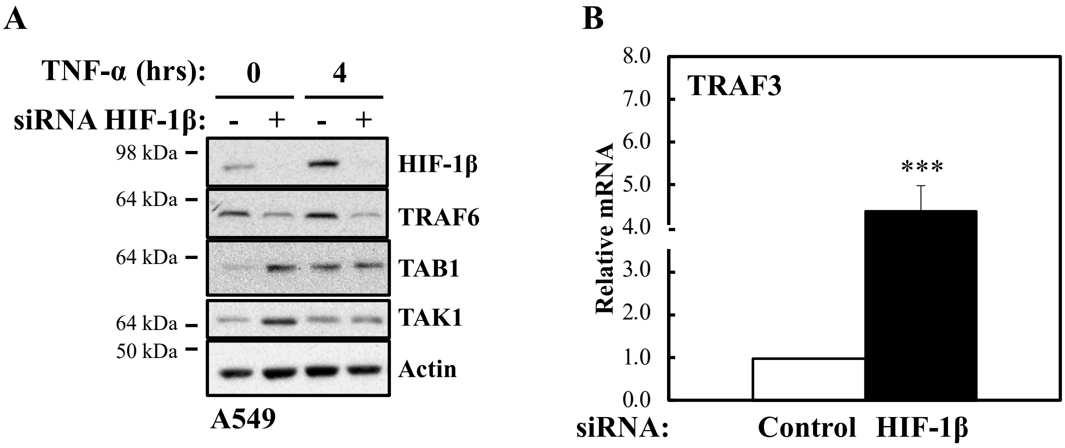

Sup. Fig. S4. HIF-1β is required for TRAF6 protein levels in A549 cells. A. A549 cells were transfected with control or HIF-1β siRNA oligonucleotides before treatment with 10ng/mL TNF-α for 4 hours prior to lysis and Western blot analysis. Actin was used as a loading control. **B.** HeLa cells were transfected with control or HIF-1β siRNA oligonucleotides prior to lysis and RNA extraction. Following cDNA synthesis, qPCR was performed using the TRAF3 primers. Graphs depict mean and SEM of a minimum of three independent biological experiments. Student t test analysis was performed and significance determined as follows: ***$p<0.001$.

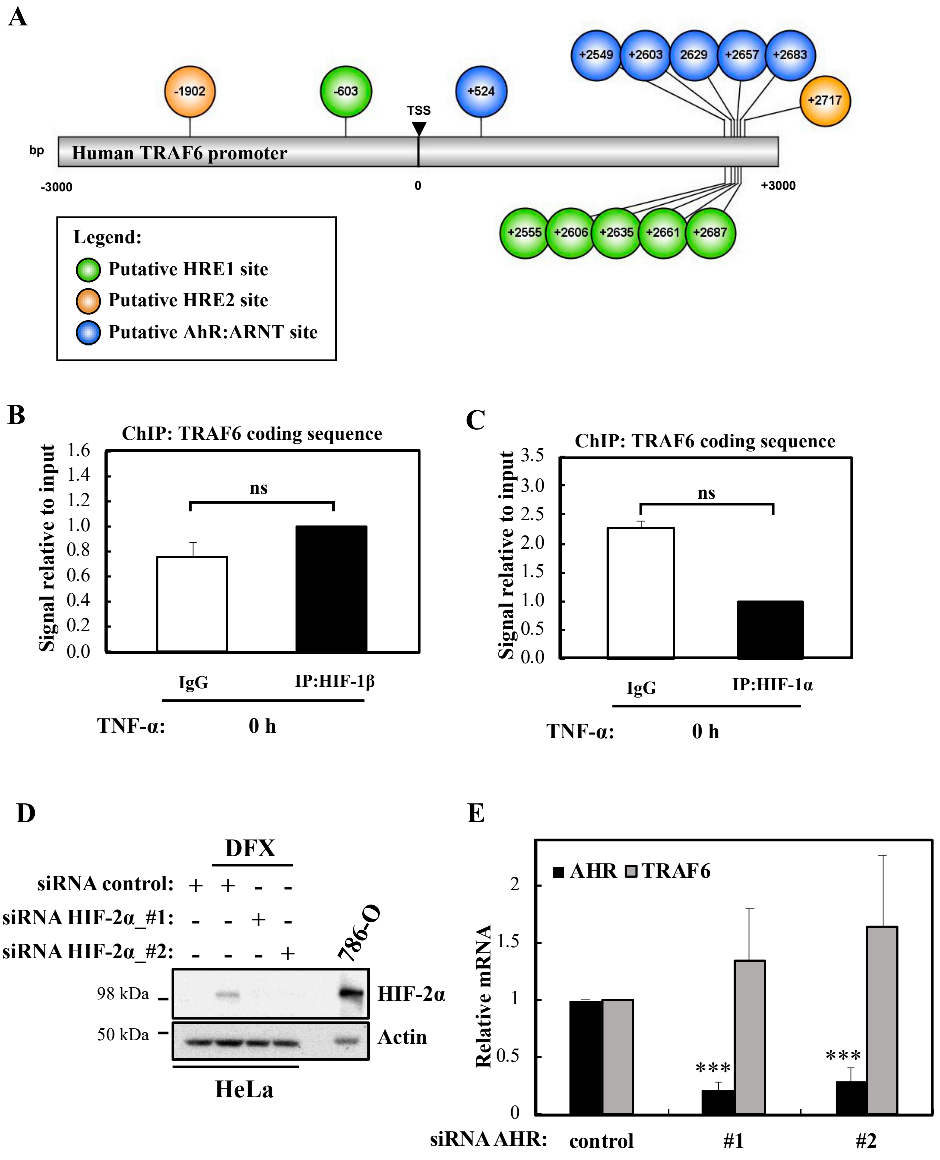

Sup. Fig. S5. TRAF6 gene contain putative HIF-1β binding sites. A. Schematic diagram of the TRAF6 gene, highlighting the putative HRE1 (5’-GCGTG-3’), HRE2 (5’-ACGTG-3’), and AHR/HIF-1β binding sites (5’-CCACGCTTCC-3’, 5’-ACACGCGCGT-3’, and 5’-ACGCGCGTGT-3’) relative to a promoter region including 3000 bp downstream and upstream TRAF6 transcription start site (TSS). Analysis was performed using the ALGGEN PROMO software tool. **B.** ChIP for HIF-1β was performed in lysates derived from HeLa cells. Occupancy at control coding regions of the TRAF6 gene was analysed by qPCR. Rabbit IgG was used as antibody control. Graph depicts mean and SEM from 3 independent biological experiments. Student t test was performed, and significance determined as follows: ns = not significant. **C**. ChIP for HIF-1α was performed in lysates derived from HeLa cells. Occupancy at control regions of the TRAF6 gene was analysed by qPCR. Rabbit IgG was used as antibody control. Graph depicts mean and SEM from 3 independent biological experiments. Student t

test was performed, and significance indicated as follows: ns = not significant. **D.** HeLa cells were transfected with control or HIF-2α siRNA oligonucleotides before treatment with 200μM Desferoxamine (DFX) for 16 hours prior to lysis and Western blot analysis. Actin was used as a loading control. 786-O cell lysates were used as a positive control for the HIF-2α antibody. **E.** HeLa cells were transfected with control or AHR siRNA oligonucleotides prior to lysis and RNA extraction. Following cDNA synthesis, qPCR was performed using the specific primers indicated. Graphs depict mean and SEM of a minimum of three independent biological experiments. One way ANOVA analysis was performed and significance determined as follows: ***p<0.001.

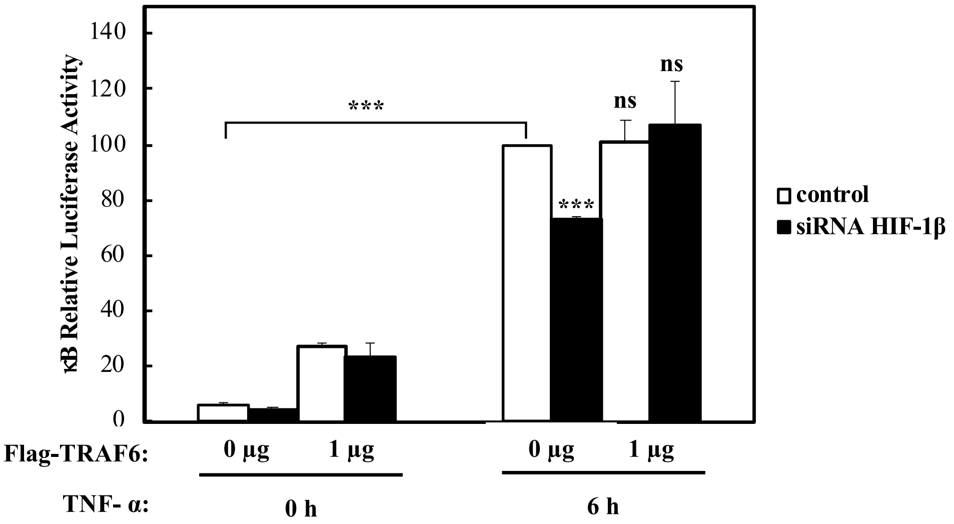

Sup. Fig. S6. Exogenous TRAF6 rescues the effects of HIF-1β depletion on NF-κB luciferase activity. HeLa-κB luciferase cells were transfected with control or TRAF6 plasmids, prior to transfection with control or HIF-1β oligonucleotides. Cells were also treated with 10ng/mL TNF-α prior to luciferase activity measurements. All the values were normalised to the control samples over-expressing control plasmid and treated with TNF-α. The graph depicts average and SEM determined from four independent biological experiments. Student t-test analysis was performed, and significance determined as follows: ns= not significant, *** p < 0.001.

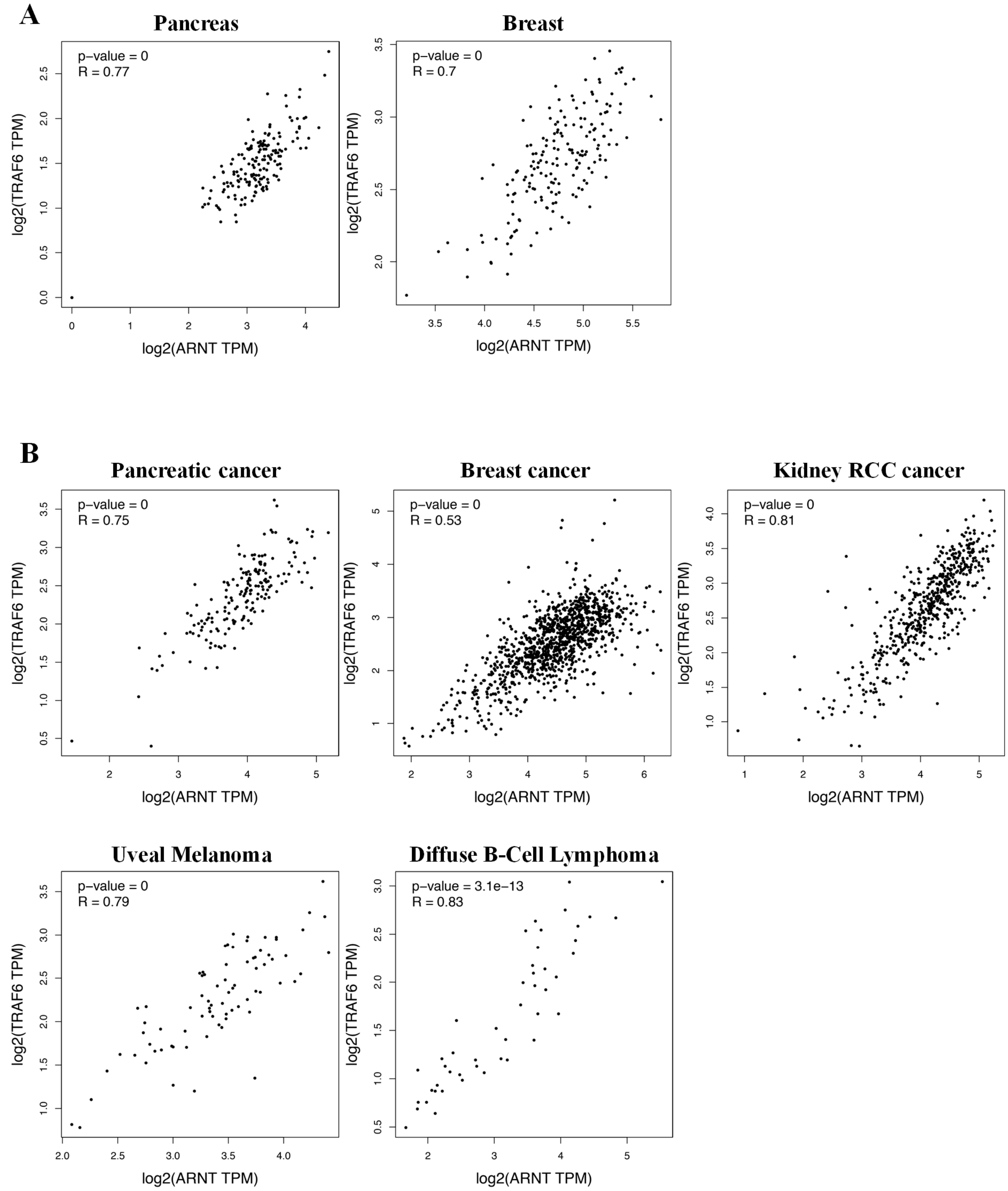

Sup. Fig. S7. HIF-1β and TRAF6 mRNA are strongly correlated in human samples derived from specific tissues. A. Analysis of HIF-1β and TRAF6 mRNA levels in human samples derived from pancreas and breast and analysed through RNA-sequencing. For this analysis the GEPIA 2.0 software tool was used. Each dot represents an individual sample. R-indicates correlation level. **B.** Analysis of HIF-1β and TRAF6 mRNA levels in human samples derived from cancer patients for the indicated types and subject to RNA-sequencing. The

GEPIA 2.0 software tool was used. Each dot represents an individual sample. R-indicates correlation level.
